# 1,1′-[(5-Hy­droxy­methyl-1,3-phenyl­ene)bis­(methyl­ene)]dipyridin-4(1*H*)-one monohydrate

**DOI:** 10.1107/S1600536811024809

**Published:** 2011-06-30

**Authors:** José A. Fernandes, Manuela E. L. Lago, Sandrina Silva, João P. C. Tomé, José A. S. Cavaleiro, Filipe A. Almeida Paz

**Affiliations:** a Department of Chemistry, University of Aveiro, CICECO, 3810-193 Aveiro, Portugal; b Department of Chemistry, University of Aveiro, QOPNA, 3810-193 Aveiro, Portugal

## Abstract

The asymmetric unit of the title compound, C_19_H_18_N_2_O_3_, comprises a whole organic dipyridinone mol­ecule plus a water mol­ecule of crystallization. The planes of the pyridinone rings are approximately perpendicular with the plane of the central aromatic ring [dihedral angles = 80.68 (8) and 83.65 (8)°]. The C—O bond of the hy­droxy group subtends an angle of 31.71 (10)° with the plane through the central aromatic ring. The crystal packing is mediated by the presence of several O—H⋯O hydrogen-bonding inter­actions and while the water mol­ecules form a *C*_2_^1^(4) chain parallel to the *c* axis of the unit cell, the pendant hy­droxy groups are engaged in O—H⋯O=C hydrogen bonds described by a *C*_1_^1^(12) graph-set motif which runs parallel to the *a* axis.

## Related literature

For previous reports on the design and synthesis of mol­ecules based on a mesitylene core, see: Reger *et al.* (2010 ▶); Podyachev *et al.* (2006 ▶); Spiccia *et al.*(1997 ▶); Newkome *et al.* (1986 ▶); Berl *et al.*, (2002 ▶). For the crystal structure and vibrational features of the precursor, 1,3,5-tris­(bromo­meth­yl)benzene, see: Fernandes *et al.* (2011 ▶). For a systematization of the graph-set notation for hydrogen-bonded aggregates, see: Grell *et al.* (1999 ▶). 
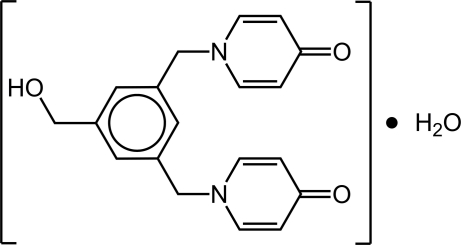


## Experimental

### 

#### Crystal data

C_19_H_18_N_2_O_3_·H_2_O
*M* *_r_* = 340.37
Monoclinic, 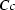

*a* = 12.2215 (8) Å
*b* = 14.1521 (10) Å
*c* = 10.3326 (7) Å
β = 114.720 (3)°
*V* = 1623.36 (19) Å^3^
*Z* = 4
Mo *K*α radiation
μ = 0.10 mm^−1^
*T* = 180 K
0.16 × 0.10 × 0.10 mm

#### Data collection

Bruker X8 KappaCCD APEXII diffractometer
Absorption correction: multi-scan (*SADABS*; Sheldrick, 1997 ▶) *T*_min_ = 0.984, *T*_max_ = 0.990
79090 measured reflections
2188 independent reflections
2103 reflections with *I* > 2σ(*I*)
*R*_int_ = 0.044

#### Refinement

*R*[*F* ^2^ > 2σ(*F* ^2^)] = 0.030
*wR*(*F* ^2^) = 0.077
*S* = 1.07
2188 reflections
233 parameters
5 restraints
H atoms treated by a mixture of independent and constrained refinement
Δρ_max_ = 0.26 e Å^−3^
Δρ_min_ = −0.20 e Å^−3^

### 

Data collection: *APEX2* (Bruker, 2006 ▶); cell refinement: *SAINT-Plus* (Bruker, 2005 ▶); data reduction: *SAINT-Plus*; program(s) used to solve structure: *SHELXTL* (Sheldrick, 2008 ▶); program(s) used to refine structure: *SHELXTL*; molecular graphics: *DIAMOND* (Brandenburg, 2009 ▶); software used to prepare material for publication: *SHELXTL*.

## Supplementary Material

Crystal structure: contains datablock(s) global, I. DOI: 10.1107/S1600536811024809/tk2759sup1.cif

Structure factors: contains datablock(s) I. DOI: 10.1107/S1600536811024809/tk2759Isup2.hkl

Supplementary material file. DOI: 10.1107/S1600536811024809/tk2759Isup3.cml

Additional supplementary materials:  crystallographic information; 3D view; checkCIF report

## Figures and Tables

**Table 1:** Hydrogen-bond geometry (Å, °)

*D*—H⋯*A*	*D*—H	H⋯*A*	*D*⋯*A*	*D*—H⋯*A*
O1*W*—H1*X*⋯O3^i^	0.94 (1)	1.93 (1)	2.842 (2)	164 (2)
O1*W*—H1*Y*⋯O3^ii^	0.94 (1)	2.03 (1)	2.973 (2)	174 (2)
O1—H1⋯O2^iii^	0.84	1.87	2.6814 (19)	162

Symmetry codes: (i) 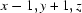; (ii) 
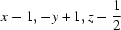
; (iii) 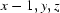.

**Fig. 1.**
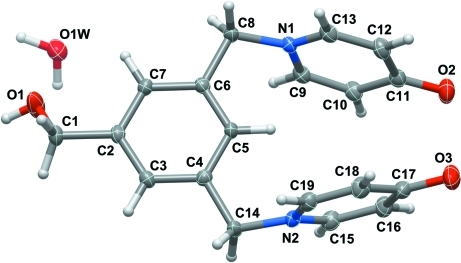
Asymmetric unit of the title compound showing the labeling scheme for all non-hydrogen atoms which are represented as thermal ellipsoids drawn at the 50% probability level. Hydrogen atoms are represented as small spheres with arbitrary radii.

**Fig. 2.**
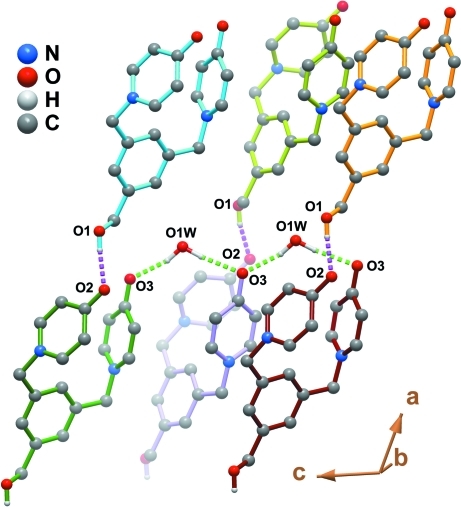
Detailed view of the hydrogen bonding interactions present in the crystal structure of the title compound. For clarity, hydrogen atoms which are not involved in hydrogen bonding interactions have been omitted, the six organic molecules of the title compound have been represented in different color and symmetry codes associated with symmetry-generated atoms have also been omitted. See Table 1 for details on the geometry of the hydrogen bonding interactions. O—H···O hydrogen bonding interactions involving the water molecules of crystallization are represented as dashed green lines while those connecting adjacent molecular units are drawn as pink dashed lines.

**Fig. 3.**
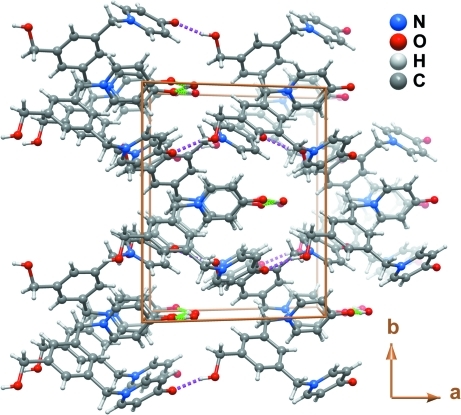
Crystal packing of the title compound viewed in perspective along the [001] direction of the unit cell. O—H···O hydrogen bonding interactions involving the water molecules of crystallization are represented as dashed green lines while those connecting adjacent molecular units are drawn as pink dashed lines.

## supplementary crystallographic information

### Comment

1,3,5-Tris(bromomethyl)benzene has been systematically employed in the preparation of many branched (Reger *et al.*, 2010; Podyachev *et al.*, 2006; Spiccia *et al.*, 1997) or dendritic molecules (Newkome *et al.*, 1986; Berl *et al.*, 2002) with a mesitylene unit comprising the core. Our research groups have also been using this molecule as a versatile template and recently we reported its crystal structure and detailed vibrational features (Fernandes *et al.*, 2011). During our research efforts with this molecule we have isolated the title compound, C_19_H_18_N_2_O_3_^.^H_2_O, as a secondary product. The title compound was obtained by the nucleophilic substitution of two bromo atoms of 1,3,5-tris(bromomethyl)benzene by 4-hydroxypyridine. Due to tautomeric equilibrium of this latter reagent, the nucleophilic attack occurred *via* the nitrogen atom and not *via* the oxygen. Spontaneous oxidation gave rise to the title compound whose crystal structure we wish to report here.

The asymmetric unit of the title compound (I) comprises a whole molecular unit, C_19_H_18_N_2_O_3_, and a water molecule of crystallization as depicted in Figure 1. The pyridone rings are almost planar (largest observed deviations of about 0.013 and 0.009 Å), and are approximately perpendicular to the plane of the central aromatic ring (dihedral angles of 80.68 (8) and 83.65 (8) °). The C—O bond belonging to the terminal hydroxy group subtends an angle of 31.71 (10)° with the plane of the central aromatic ring.

The presence of polar O—H bonds and the C═O moieties located in opposite positions of the organic moiety permits the existence of several hydrogen bonding interactions whose geometric details are tabulated in Table 1. On the one hand, the two hydrogen atoms of the water molecule of crystallization interact with neighbouring O3 atoms from adjacent organic molecules, leading to the formation of a supramolecular polymeric chain parallel to the *c*-axis of the unit cell, describing a *C^1^_2_(4)* graph set motif (Grell *et al.*, 1999), Figs 2 and 3. On the other hand, the pendant hydroxy groups are engaged in O—H···O═C hydrogen bonds, Figs 2 and 3, which permits a direct connection between adjacent molecular units. In addition, this connection leads to the formation of a *C^1^_1_(12)* graph set motif parallel to the *a*-axis.

### Experimental

All chemicals were purchased from commercial sources and were used without further purification: 4-hydroxypyridine (Fluka, 95%); potassium carbonate (Vaz Pereira); 1,3,5-tris(bromomethyl)benzene (Sigma-Aldrich, 97%).

An excess of potassium carbonate (200 mg, 1.45 mmol) was added to a solution of 4-hydroxypyridine (52.8 mg, 0.56 mmol) in dry dimethylformamide (DMF, 2.5 mL). The resulting mixture was stirred under N_2_ for 30 minutes at ambient temperature. This mixture was then added drop wise to a DMF (2.5 mL) solution of 1,3,5-tris(bromomethyl)benzene (100 mg, 0.28 mmol). The new reaction mixture was maintained under constant magnetic stirring for 3 h under N_2_. The resulting products were precipitated by the addition of diethyl ether, filtered and purified by column chromatography (silica gel) using a mixture of THF/MeOH (1:2) as eluent. The title compound was crystallized from a mixture of CHCl_3_/MeOH (95:5). Yield: 25%.

^1^H NMR (300 MHz, CDCl_3_/MeOD): δ 4.51 (s, 2H, CH_2_OH), 4.93 (s, 4H, CH_2_N), 6.31 (d, *J_o_*_-m_= 7.6 Hz, 4H, *m*-H), 6.89 (s, 1H, ArH-2), 7.10 (s, 2H, ArH-4, 6), 7.44 (d, *J_o_*_-m_= 7.6 Hz, 4H, *o*-H).

^13^C NMR (75 MHz, CDCl_3_/MeOD): δ 63.2 (CH_2_N), 67.9 (CH_2_OH), 118.2 (NCH_2_CH_2_CO), 125.3 (ArC-2), 126.2 (ArC-4, 6), 136.2 (ArC-1, 3), 141.0 (NCH_2_CH_2_CO), 144.6 (ArC-5), 179.4 (CO).

### Refinement

Hydrogen atoms bound to carbon and the hydroxy group were placed at their idealized positions and were included in the final structural model in riding-motion approximation with C—H = 0.95 Å (aromatic C—H), C—H = 0.99 Å (—CH_2_—) and O—H = 0.84 Å (—OH). The isotropic thermal displacement parameters associated with these atoms were fixed at 1.2 (for those bound to carbon) or 1.5 (for that associated with the hydroxy group) ×*U*_eq_ of the parent atom. Hydrogen atoms associated with the water molecule of crystallization were directly located from difference Fourier maps and were included in the structure with the O—H and H···H distances restrained to 0.95 (1) and 1.55 (1) Å, respectively, and *U*_iso_(H)=1.5×*U*_eq_(O). In the absence of significant anomalous scattering effects, 2147 Friedel pairs were averaged in the final refinement.

### Crystal data

**Table:**

C_19_H_18_N_2_O_3_·H_2_O	*F*(000) = 720
*M**_r_* = 340.37	*D*_x_ = 1.393 Mg m^−^^3^
Monoclinic, *C**c*	Mo *K*α radiation, λ = 0.71073 Å
Hall symbol: C -2yc	Cell parameters from 9702 reflections
*a* = 12.2215 (8) Å	θ = 2.6–29.1°
*b* = 14.1521 (10) Å	µ = 0.10 mm^−^^1^
*c* = 10.3326 (7) Å	*T* = 180 K
β = 114.720 (3)°	Block, colourless
*V* = 1623.36 (19) Å^3^	0.16 × 0.10 × 0.10 mm
*Z* = 4	

### Data collection

**Table:**

Bruker X8 KappaCCD APEXII diffractometer	2188 independent reflections
Radiation source: fine-focus sealed tube	2103 reflections with *I* > 2σ(*I*)
graphite	*R*_int_ = 0.044
ω and φ scans	θ_max_ = 29.1°, θ_min_ = 3.6°
Absorption correction: multi-scan (*SADABS*; Sheldrick, 1997)	*h* = −16→16
*T*_min_ = 0.984, *T*_max_ = 0.990	*k* = −19→19
79090 measured reflections	*l* = −14→14

### Refinement

**Table:**

Refinement on *F*^2^	Secondary atom site location: difference Fourier map
Least-squares matrix: full	Hydrogen site location: inferred from neighbouring sites
*R*[*F*^2^ > 2σ(*F*^2^)] = 0.030	H atoms treated by a mixture of independent and constrained refinement
*wR*(*F*^2^) = 0.077	*w* = 1/[σ^2^(*F*_o_^2^) + (0.0434*P*)^2^ + 0.5107*P*] where *P* = (*F*_o_^2^ + 2*F*_c_^2^)/3
*S* = 1.07	(Δ/σ)_max_ < 0.001
2188 reflections	Δρ_max_ = 0.26 e Å^−^^3^
233 parameters	Δρ_min_ = −0.20 e Å^−^^3^
5 restraints	Absolute structure: nd
Primary atom site location: structure-invariant direct methods	

### Special details

**Table:**

Geometry. All e.s.d.'s (except the e.s.d. in the dihedral angle between two l.s. planes) are estimated using the full covariance matrix. The cell e.s.d.'s are taken into account individually in the estimation of e.s.d.'s in distances, angles and torsion angles; correlations between e.s.d.'s in cell parameters are only used when they are defined by crystal symmetry. An approximate (isotropic) treatment of cell e.s.d.'s is used for estimating e.s.d.'s involving l.s. planes.
Refinement. Refinement of *F*^2^ against ALL reflections. The weighted *R*-factor *wR* and goodness of fit *S* are based on *F*^2^, conventional *R*-factors *R* are based on *F*, with *F* set to zero for negative *F*^2^. The threshold expression of *F*^2^ > σ(*F*^2^) is used only for calculating *R*-factors(gt) *etc*. and is not relevant to the choice of reflections for refinement. *R*-factors based on *F*^2^ are statistically about twice as large as those based on *F*, and *R*- factors based on ALL data will be even larger.

### Fractional atomic coordinates and isotropic or equivalent isotropic displacement parameters (Å^2^)

**Table:**

	*x*	*y*	*z*	*U*_iso_*/*U*_eq_	
N1	0.92401 (12)	0.28077 (10)	0.65493 (14)	0.0187 (3)	
N2	0.79193 (12)	0.00864 (10)	0.34079 (15)	0.0196 (3)	
O1	0.38592 (12)	0.23425 (10)	0.59461 (18)	0.0387 (4)	
H1	0.3109	0.2418	0.5601	0.058*	
O2	1.15708 (12)	0.29372 (10)	0.46529 (15)	0.0317 (3)	
O3	1.14843 (12)	−0.01091 (12)	0.42158 (16)	0.0384 (4)	
C1	0.41238 (14)	0.14197 (11)	0.56300 (19)	0.0216 (3)	
H1A	0.3467	0.1195	0.4735	0.026*	
H1B	0.4184	0.0982	0.6405	0.026*	
C2	0.53025 (14)	0.14250 (10)	0.54728 (17)	0.0175 (3)	
C3	0.62390 (14)	0.20213 (11)	0.63200 (17)	0.0181 (3)	
H3	0.6132	0.2432	0.6985	0.022*	
C4	0.73306 (14)	0.20150 (11)	0.61923 (16)	0.0174 (3)	
C5	0.74897 (14)	0.14000 (11)	0.52290 (17)	0.0182 (3)	
H5	0.8241	0.1382	0.5160	0.022*	
C6	0.65561 (14)	0.08125 (11)	0.43687 (17)	0.0179 (3)	
C7	0.54605 (14)	0.08265 (11)	0.44961 (17)	0.0185 (3)	
H7	0.4821	0.0425	0.3913	0.022*	
C8	0.83252 (14)	0.26795 (12)	0.71127 (18)	0.0210 (3)	
H8A	0.7972	0.3301	0.7161	0.025*	
H8B	0.8714	0.2423	0.8092	0.025*	
C9	0.89558 (15)	0.33240 (12)	0.53364 (18)	0.0217 (3)	
H9	0.8219	0.3665	0.4953	0.026*	
C10	0.97013 (15)	0.33616 (12)	0.46626 (19)	0.0231 (3)	
H10	0.9469	0.3717	0.3809	0.028*	
C11	1.08376 (14)	0.28714 (12)	0.52208 (18)	0.0222 (3)	
C12	1.10808 (15)	0.23253 (12)	0.64795 (19)	0.0247 (4)	
H12	1.1806	0.1971	0.6892	0.030*	
C13	1.02879 (15)	0.23077 (12)	0.70921 (17)	0.0221 (3)	
H13	1.0471	0.1936	0.7922	0.027*	
C14	0.66668 (14)	0.01871 (12)	0.32359 (19)	0.0229 (3)	
H14A	0.6339	−0.0446	0.3278	0.027*	
H14B	0.6175	0.0458	0.2285	0.027*	
C15	0.84155 (16)	0.07180 (13)	0.28150 (19)	0.0245 (3)	
H15	0.7922	0.1199	0.2209	0.029*	
C16	0.96049 (16)	0.06788 (13)	0.30689 (19)	0.0265 (4)	
H16	0.9922	0.1132	0.2639	0.032*	
C17	1.03820 (16)	−0.00342 (13)	0.39709 (19)	0.0253 (4)	
C18	0.98150 (16)	−0.06684 (13)	0.45843 (19)	0.0265 (4)	
H18	1.0280	−0.1153	0.5208	0.032*	
C19	0.86260 (16)	−0.05917 (11)	0.42929 (18)	0.0229 (3)	
H19	0.8281	−0.1024	0.4720	0.027*	
O1W	0.26763 (13)	0.99519 (12)	0.23725 (16)	0.0365 (3)	
H1X	0.216 (2)	0.998 (2)	0.283 (2)	0.055*	
H1Y	0.224 (2)	0.999 (2)	0.1375 (11)	0.055*	

### Atomic displacement parameters (Å^2^)

**Table:**

	*U*^11^	*U*^22^	*U*^33^	*U*^12^	*U*^13^	*U*^23^
N1	0.0158 (6)	0.0206 (6)	0.0200 (6)	−0.0034 (5)	0.0077 (5)	−0.0024 (5)
N2	0.0178 (6)	0.0221 (6)	0.0206 (6)	0.0011 (5)	0.0096 (5)	−0.0030 (5)
O1	0.0209 (6)	0.0327 (7)	0.0641 (10)	0.0020 (5)	0.0195 (7)	−0.0139 (7)
O2	0.0235 (6)	0.0414 (7)	0.0351 (7)	0.0019 (6)	0.0172 (6)	0.0058 (6)
O3	0.0206 (6)	0.0613 (10)	0.0345 (8)	0.0042 (6)	0.0126 (6)	0.0033 (7)
C1	0.0175 (7)	0.0236 (7)	0.0267 (8)	0.0006 (6)	0.0121 (6)	0.0023 (6)
C2	0.0153 (6)	0.0193 (7)	0.0188 (7)	0.0018 (6)	0.0080 (6)	0.0048 (6)
C3	0.0183 (7)	0.0190 (7)	0.0190 (7)	0.0012 (6)	0.0099 (6)	−0.0004 (6)
C4	0.0159 (7)	0.0187 (7)	0.0173 (7)	−0.0005 (5)	0.0064 (6)	0.0004 (5)
C5	0.0142 (6)	0.0215 (7)	0.0202 (7)	−0.0005 (6)	0.0085 (6)	−0.0028 (6)
C6	0.0184 (7)	0.0180 (6)	0.0180 (7)	0.0012 (5)	0.0081 (6)	−0.0003 (5)
C7	0.0157 (7)	0.0182 (7)	0.0206 (7)	−0.0012 (5)	0.0067 (6)	0.0008 (6)
C8	0.0178 (7)	0.0248 (8)	0.0227 (7)	−0.0043 (6)	0.0107 (6)	−0.0064 (6)
C9	0.0178 (7)	0.0206 (7)	0.0241 (8)	0.0000 (6)	0.0063 (6)	0.0008 (6)
C10	0.0203 (7)	0.0246 (8)	0.0233 (7)	−0.0006 (6)	0.0081 (6)	0.0045 (6)
C11	0.0196 (8)	0.0227 (8)	0.0250 (8)	−0.0034 (6)	0.0099 (7)	−0.0025 (6)
C12	0.0192 (8)	0.0242 (8)	0.0294 (9)	0.0021 (6)	0.0090 (7)	0.0041 (7)
C13	0.0193 (7)	0.0214 (7)	0.0231 (8)	−0.0008 (6)	0.0063 (6)	0.0024 (6)
C14	0.0159 (7)	0.0287 (8)	0.0248 (8)	−0.0021 (6)	0.0092 (6)	−0.0092 (7)
C15	0.0263 (8)	0.0250 (8)	0.0237 (8)	0.0035 (6)	0.0120 (7)	0.0032 (6)
C16	0.0258 (8)	0.0313 (9)	0.0260 (9)	−0.0011 (7)	0.0144 (7)	0.0028 (7)
C17	0.0224 (8)	0.0328 (9)	0.0217 (8)	0.0012 (7)	0.0103 (7)	−0.0026 (7)
C18	0.0261 (9)	0.0273 (8)	0.0269 (8)	0.0071 (7)	0.0118 (7)	0.0055 (7)
C19	0.0271 (8)	0.0200 (7)	0.0246 (8)	−0.0001 (6)	0.0139 (7)	0.0004 (6)
O1W	0.0245 (6)	0.0518 (9)	0.0308 (7)	−0.0028 (6)	0.0091 (6)	−0.0021 (6)

### Geometric parameters (Å, °)

**Table:**

N1—C13	1.362 (2)	C7—H7	0.9500
N1—C9	1.364 (2)	C8—H8A	0.9900
N1—C8	1.472 (2)	C8—H8B	0.9900
N2—C19	1.357 (2)	C9—C10	1.359 (2)
N2—C15	1.361 (2)	C9—H9	0.9500
N2—C14	1.472 (2)	C10—C11	1.440 (2)
O1—C1	1.416 (2)	C10—H10	0.9500
O1—H1	0.8400	C11—C12	1.433 (2)
O2—C11	1.263 (2)	C12—C13	1.361 (2)
O3—C17	1.267 (2)	C12—H12	0.9500
C1—C2	1.516 (2)	C13—H13	0.9500
C1—H1A	0.9900	C14—H14A	0.9900
C1—H1B	0.9900	C14—H14B	0.9900
C2—C7	1.391 (2)	C15—C16	1.366 (2)
C2—C3	1.397 (2)	C15—H15	0.9500
C3—C4	1.394 (2)	C16—C17	1.431 (3)
C3—H3	0.9500	C16—H16	0.9500
C4—C5	1.396 (2)	C17—C18	1.433 (3)
C4—C8	1.516 (2)	C18—C19	1.360 (2)
C5—C6	1.391 (2)	C18—H18	0.9500
C5—H5	0.9500	C19—H19	0.9500
C6—C7	1.399 (2)	O1W—H1X	0.936 (10)
C6—C14	1.518 (2)	O1W—H1Y	0.943 (10)
			
C13—N1—C9	119.41 (14)	C10—C9—N1	121.57 (15)
C13—N1—C8	120.81 (14)	C10—C9—H9	119.2
C9—N1—C8	119.05 (14)	N1—C9—H9	119.2
C19—N2—C15	119.32 (14)	C9—C10—C11	121.12 (15)
C19—N2—C14	119.10 (14)	C9—C10—H10	119.4
C15—N2—C14	121.23 (15)	C11—C10—H10	119.4
C1—O1—H1	109.5	O2—C11—C12	122.89 (16)
O1—C1—C2	109.83 (13)	O2—C11—C10	122.09 (15)
O1—C1—H1A	109.7	C12—C11—C10	115.00 (14)
C2—C1—H1A	109.7	C13—C12—C11	120.93 (15)
O1—C1—H1B	109.7	C13—C12—H12	119.5
C2—C1—H1B	109.7	C11—C12—H12	119.5
H1A—C1—H1B	108.2	C12—C13—N1	121.92 (15)
C7—C2—C3	119.77 (14)	C12—C13—H13	119.0
C7—C2—C1	120.07 (14)	N1—C13—H13	119.0
C3—C2—C1	120.15 (14)	N2—C14—C6	112.76 (13)
C4—C3—C2	120.17 (14)	N2—C14—H14A	109.0
C4—C3—H3	119.9	C6—C14—H14A	109.0
C2—C3—H3	119.9	N2—C14—H14B	109.0
C3—C4—C5	119.71 (14)	C6—C14—H14B	109.0
C3—C4—C8	118.98 (13)	H14A—C14—H14B	107.8
C5—C4—C8	121.31 (13)	N2—C15—C16	121.70 (16)
C6—C5—C4	120.45 (14)	N2—C15—H15	119.1
C6—C5—H5	119.8	C16—C15—H15	119.1
C4—C5—H5	119.8	C15—C16—C17	121.11 (16)
C5—C6—C7	119.53 (14)	C15—C16—H16	119.4
C5—C6—C14	121.83 (14)	C17—C16—H16	119.4
C7—C6—C14	118.56 (14)	O3—C17—C16	123.34 (17)
C2—C7—C6	120.35 (14)	O3—C17—C18	121.98 (17)
C2—C7—H7	119.8	C16—C17—C18	114.68 (15)
C6—C7—H7	119.8	C19—C18—C17	121.44 (16)
N1—C8—C4	111.76 (13)	C19—C18—H18	119.3
N1—C8—H8A	109.3	C17—C18—H18	119.3
C4—C8—H8A	109.3	N2—C19—C18	121.72 (15)
N1—C8—H8B	109.3	N2—C19—H19	119.1
C4—C8—H8B	109.3	C18—C19—H19	119.1
H8A—C8—H8B	107.9	H1X—O1W—H1Y	111.4 (15)
			
O1—C1—C2—C7	−145.77 (15)	C9—C10—C11—O2	−176.23 (17)
O1—C1—C2—C3	35.1 (2)	C9—C10—C11—C12	2.4 (2)
C7—C2—C3—C4	−0.2 (2)	O2—C11—C12—C13	177.05 (17)
C1—C2—C3—C4	178.93 (14)	C10—C11—C12—C13	−1.6 (2)
C2—C3—C4—C5	−0.9 (2)	C11—C12—C13—N1	−0.4 (3)
C2—C3—C4—C8	179.21 (14)	C9—N1—C13—C12	1.7 (2)
C3—C4—C5—C6	1.8 (2)	C8—N1—C13—C12	171.78 (15)
C8—C4—C5—C6	−178.39 (15)	C19—N2—C14—C6	85.84 (19)
C4—C5—C6—C7	−1.4 (2)	C15—N2—C14—C6	−87.4 (2)
C4—C5—C6—C14	175.51 (15)	C5—C6—C14—N2	15.0 (2)
C3—C2—C7—C6	0.6 (2)	C7—C6—C14—N2	−168.12 (14)
C1—C2—C7—C6	−178.55 (15)	C19—N2—C15—C16	1.1 (2)
C5—C6—C7—C2	0.2 (2)	C14—N2—C15—C16	174.26 (16)
C14—C6—C7—C2	−176.82 (15)	N2—C15—C16—C17	0.2 (3)
C13—N1—C8—C4	−98.31 (17)	C15—C16—C17—O3	178.65 (18)
C9—N1—C8—C4	71.82 (18)	C15—C16—C17—C18	−1.2 (3)
C3—C4—C8—N1	−161.86 (14)	O3—C17—C18—C19	−178.78 (17)
C5—C4—C8—N1	18.3 (2)	C16—C17—C18—C19	1.1 (3)
C13—N1—C9—C10	−0.8 (2)	C15—N2—C19—C18	−1.2 (2)
C8—N1—C9—C10	−171.09 (15)	C14—N2—C19—C18	−174.54 (16)
N1—C9—C10—C11	−1.3 (3)	C17—C18—C19—N2	0.1 (3)

### Hydrogen-bond geometry (Å, °)

**Table:**

*D*—H···*A*	*D*—H	H···*A*	*D*···*A*	*D*—H···*A*
O1W—H1X···O3^i^	0.94 (1)	1.93 (1)	2.842 (2)	164 (2)
O1W—H1Y···O3^ii^	0.94 (1)	2.03 (1)	2.973 (2)	174 (2)
O1—H1···O2^iii^	0.84	1.87	2.6814 (19)	162

Symmetry codes: (i) *x*−1, *y*+1, *z*; (ii) *x*−1, −*y*+1, *z*−1/2; (iii) *x*−1, *y*, *z*.
